# Future Applications of Biotechnology to the Energy Industry

**DOI:** 10.3389/fmicb.2016.00086

**Published:** 2016-02-04

**Authors:** John J. Kilbane

**Affiliations:** ^1^Intertek Westport Technology CenterHouston, TX, USA; ^2^Biological, Chemical and Physical Sciences, Illinois Institute of TechnologyChicago, IL, USA

**Keywords:** biotechnology, biofuels, energy, petroleum, biomethanation, future

There has been a dramatic growth in the production of biofuels in recent times. Global biofuel production tripled between 2000 and 2007, and biofuels accounted for about 1.6% of global transportation fuel in 2012 (International Energy Agency). At the time of this writing, 2015, ethanol production is by far the greatest contribution by biotechnology to energy production, with revenue accounting for $40.9 billion worldwide in 2014 vs. $3.8 billion for biodiesel and $0.019 billion for bio-methane. It is logical to imagine the future contribution of biotechnology to world energy production may increase not only in the area of biofuel production, but also in petroleum production, petroleum upgrading, biogas production, chemical production, crop improvement, bioremediation, microbiologically influenced corrosion, space travel, and other topics. However, the future contributions of biotechnology to the energy industry are not only influenced by technical advances in biotechnology, but also by the price of fossil fuels, the development of renewable energy generally, politics, global population growth, and other factors. Concerns about the use of crops for food versus fuel production, environmental effects of land use related to biofuel production, decreased oil prices, ever-increasing advances in the generation and use of wind and solar energy, and political will to promote/subsidize the development of alternative energy are also influencing factors.

## Biotechnology and the fossil fuel industry

The contributions of biotechnology to the energy industry are not restricted to the production of biofuels, and the microbial production of methane may well be the largest contribution in the future. From 60 to 80% of oil in geological deposits is left in place by the oil industry as it is considered to be technically and/or economically non-recoverable (Muggeridge et al., 2014). However, microbial conversion of hydrocarbons to methane could dramatically increase the amount of energy recovered. Quantification of the relative abundance of stable isotopes of carbon and hydrogen can reveal the origin of methane in geological deposits because chemical and biochemical pathways for the formation of methane have different reactivities/preferences for different isotopes. It is estimated that 20–40% of methane in oil and gas reservoirs is of microbial origin (Katz, 2011) and most of that is derived from the conversion of carbon dioxide into methane. Similarly, the presence of biologically produced methane in coal deposits demonstrates that biotechnology can also aid in the recovery of energy from coal (Cheung et al., 2010). If depleted/uneconomical oil and coal deposits were treated in an appropriate manner it is conceivable that the residual hydrocarbon value in those deposits could be recovered at an accelerated rate through the use of CO2 injection and biomethanation. This approach would allow multiple cycles of CO2 injection and methane harvest to occur, rather than a one-time injection and disposal of CO2.

Therefore, the potential exists to employ biotechnology to convert the residual hydrocarbons in depleted oil wells and coal deposits into methane and recover a far greater percentage of the energy content in a reasonable time frame while simultaneously reducing the amount of CO2 released to the atmosphere (Geig et al., 2008).

Biotechnology can be used to upgrade petroleum and coal by removing undesirable elements/components such as sulfur, nitrogen, metals, and ash and by reducing viscosity. Bioprocessing can make oil easier/less expensive to refine and can reduce the production of air polluting gases resulting from the combustion of oil and coal (Youssef et al., 2009; Bachmann et al., 2014). However, these applications of biotechnology to the energy industry have not yet been implemented on a commercial scale, so it remains to be seen if future developments can overcome current obstacles. The chief obstacle for the implementation of any technology is cost. The biotechnology industry has previously been dominated by the production of low volumes of high value products, while the production of biofuels such as ethanol and biodiesel seek to make large volumes of products at the lowest possible cost. It is conceivable that the greatest impact of the development of biofuels will be to transform the biotechnology industry. Experience gained in the production of large volumes of low cost biofuels has the potential to dramatically increase the number and decrease the cost of products from the biotechnology industry worldwide.

## Biotechnology in space

One of the most important crew members for interstellar travel will be the biotechnologist. Space travel for extended times creates nutritional, air and water quality, medical, and other issues that can be addressed through biotechnology. Methanogenic bacteria degrade organic matter, such as excrement, and produce methane. Recycling organic waste is crucially important in space travel where living space is limited and every available resource must be utilized. The biodegradation of organic waste creates methane as well as composted soil/nutrients that can be used to grow plants and/or photosynthetic microbes that can utilize sunlight and carbon dioxide to produce oxygen and food. Methanotrophs utilize methane for growth and have been demonstrated to be a nutritionally complete food source (Overland et al., 2010), and through the use of genetic engineering it is possible to use methanotrophs to produce nearly any biotechnology industry product without using carbon sources like sugars that can be used to feed people and animals (Sharpe et al., 2007). The rapid growth and small space requirements for the growth of methanotrophs (Gilman et al., 2015) will allow a diversity of products to be made in space, and is more practical than trying to stock a space ship with every pharmaceutical/bioproduct that may be needed.

## Recycling organic waste

Just as the recycling of nutrients from waste material is important in space travel, nutrient recycling from all forms of waste will be increasingly important in the future for sustaining agricultural productivity on Earth. Sixty percent of the world's arable lands have mineral deficiencies or elemental toxicity problems (Fageria et al., 2008). Fertilizers increase the cost of food production and increasingly contribute to environmental pollution. Biotechnology, and engineering, can make great contributions to waste management to improve methane recovery from landfills and other waste, and to produce organic fertilizers to sustain agriculture.

The overwhelming majority of current ethanol production comes from sugar cane and corn that could be used for human and/or animal food. Biofuel production in the future will be increasingly derived from materials currently considered as waste. The goal of the ethanol industry is to shift to the use of agricultural wastes (lignocellulosic material) instead of sugar cane or corn for the production of ethanol (Voegle, 2013; Miller and Sorrell, 2014; Azad et al., 2015). While claims that the production of biofuel was a key cause in the doubling of the prices for rice, wheat and maize from 2005 to 2008 have been demonstrated to be false (Suzuki et al., 2015), it is crucial that the production of biofuels in the future should not compete (or seen to compete) with the production of food for people or animals, and that biofuel production should not be the cause of deforestation or any form of environmental damage.

While agricultural wastes are not food, it is first necessary to convert lignocellulosic material into simple sugars and only then can ethanol, butanol, and other biofuels be produced. Those simple sugars derived from agricultural waste could be used as human and/or animal food, but the fermentation industry is likely to need those sugars as the feedstocks to support the future production of pharmaceuticals, nutriceuticals, vitamins, enzymes, bio-plastics, enzymes, organic acids, and all the other products valued at $173 billion in 2013 made by the global biotechnology industry (Biotechnology Market Analysis and Segment Forecasts to 2020, ISBN: 978-1-68038-134-4, 2014). The same societal and political forces that influence the fuel ethanol industry to switch from the use of food crops to agricultural wastes will increasingly act on the biotechnology industry generally to make that same switch.

## Energy, the international balance of trade, and renewable fuel sources in the future

Modern society is increasingly dependent on the abundant supply of energy. Traditionally, fossil fuels have supplied the vast majority of energy and the income from fossil fuel sales and the expense from fossil fuel purchases have been the largest contributors to the economic status of countries (Wiedmann et al., 2015). If a country has fossil fuel deposits these are valuable resources for sure, but they are a mixed blessing. The exploitation of these fossil fuel resources requires capital investment and technology that may be beyond the capabilities of some countries resulting in the involvement of foreign companies, banks, workers, and political agendas in bringing these fossil fuels to the market.

In these countries the development of these fossil fuel resources brings increased revenue, but often at the expense of increased corruption, income inequality, and foreign involvement generally without the promised benefits of increased employment, technology, manufacturing, and infrastructure development (Al-Kasim et al., 2013).

Therefore, when predicting the impact of biotechnology to the energy industry in the future it is a certainty that the production of biofuels from biomass resources will increasingly contribute to the global energy supply, and that the development of renewable energy will increasingly be promoted by those countries that lack fossil fuel resources. Currently countries/communities that do not possess fossil fuel resources must spend a high percentage of their gross domestic product to import energy. This results in a negative trade balance and a huge source of debt. However, if biomass resources are available, then modern and increasingly efficient technologies can be applied to convert biomass and/or organic waste resources to energy with modest capital investment as compared with capital investments needed to produce fossil fuels (Al-Kasim et al., 2013). The investment in the development of renewable fuels from biomass and organic waste almost exclusively results in the creation of jobs in the local economy (http://www.irena.org/News/Description.aspx?NType=A&mnu=cat&PriMenuID=16&CatID=84&News_ID=407). In contrast, the development of fossil fuel resources or the production of solar or wind energy creates fewer jobs than biofuel production and frequently the fossil/solar/wind jobs are not a part of the local economy and instead usually results in a disproportionate creation of jobs in technologically affluent countries at the expense of technologically deficient countries.

The rapid development of improved technologies for the production of biofuels from readily available biomass resources, and the relatively low cost of constructing biofuel production facilities as compared with fossil fuels (Al-Kasim et al., 2013), will make biofuels highly attractive for implementation in economically disadvantaged parts of the world. Biotechnology can provide much of the power to support a modern industrial society, using readily available and easily implemented technology (Cremonez et al., 2015; Wiedmann et al., 2015). Therefore, the current international imbalance of trade will be increasingly rebalanced in the future by the production of biofuels, and other forms of renewable energy.

## Conclusions

Biotechnology can contribute to the fossil fuel industry by assisting the production of fossil fuels, upgrading fuels, bioremediation of water, soil, and air, and in the control of microbiologically influenced corrosion (MIC; Youssef et al., 2009; Bachmann et al., 2014). The application of biotechnology to increase the production of fossil fuels is mostly experimental, but the potential growth of this area is immense. Increasing the recovery of energy from depleted/uneconomical petroleum and coal deposits, particularly in combination with CO2 utilization, could be a major component of the biotechnology industry in the future. The production of liquid biofuels and methane from organic wastes has increased dramatically in recent years, but the worldwide use of these technologies has barely begun so the future will undoubtedly see exciting growth in this area. It would seem clear that biotechnology can make even greater contributions to the energy industry in the future, but some analysts conclude that the entirety of global energy can be supplied in the future using wind, water, and solar power without the use of biotechnology (Jacobson and Delucchi, 2011), so the challenge to the biotechnology industry is to continue to demonstrate relevance to the energy industry.

## Author contributions

The author confirms being the sole contributor of this work and approved it for publication.

### Conflict of interest statement

The author declares that the research was conducted in the absence of any commercial or financial relationships that could be construed as a potential conflict of interest.
